# Regional Health Disparities in Hypertension-Related Hospitalization of Hypertensive Patients: A Nationwide Population-Based Nested Case-Control Study

**DOI:** 10.3389/ijph.2023.1605495

**Published:** 2023-01-24

**Authors:** Woo-Ri Lee, Jun Hyuk Koo, Ji Yun Jeong, Min Su Kim, Ki-Bong Yoo

**Affiliations:** ^1^ Division of Cancer Control and Policy, National Cancer Control Institute, National Cancer Center, Goyang, Republic of Korea; ^2^ HIRA Research Institute, Health Insurance Review & Assessment Service (HIRA), Wonju, Republic of Korea; ^3^ Gangwon Public Health Policy Institute, Chuncheon, Republic of Korea; ^4^ Division of Health Administration, College of Software and Digital Healthcare Convergence, Yonsei University, Wonju, Republic of Korea

**Keywords:** hypertension, hypertension-related hospitalization, continuity of care, regional health disparity, nested case-control study, NHIS-NSC

## Abstract

**Objective:** This study aims to explore regional health disparities in hypertension-related hospitalizations and confirm this difference according to the states of continuity of care (COC).

**Methods:** We used the National Health Insurance Service National Sample Cohort data from 2002 to 2019. The dependent variable, hypertension-related hospitalization, included hospitalization for hypertensive diseases (I10–I13, I15), ischemic heart disease (I20–I25), and cerebrovascular disease (I60–I69). Nested case-control matching was performed according to age, sex, and income level. We compared hypertension-related hospitalization fractions in urban and rural areas by classifying them according to the state of COC and analyzed them using conditional logistic regression suitable for matched data.

**Results:** The odds of hypertension-related hospitalization of hypertensive patients were higher in the rural areas than in the urban areas; however, as the COC increased, the difference decreased. There was no change in the results according to the COC observation period.

**Conclusion:** To reduce regional health disparities, both the promotion of COC and the improvement of the quality of primary care must be achieved.

## Introduction

Access to medical care is high in South Korea compared to that in other countries. Many infrastructures are concentrated in the metropolitan areas due to the high population density [1]; therefore, medical infrastructure is also concentrated in the metropolitan area. The environment within a given community has emerged as a factor affecting the health of residents; furthermore, regional health disparities due to unbalanced allocation of medical resources are increasing [2, 3] (See Supplementary Table S1).

Globally, populations are aging rapidly, resulting in changes in the spectrum of diseases and an increase in the number of people with multiple chronic diseases [4]. In particular, South Korea is the most rapidly aging country in the world [5], and health problems are more severe in rural areas than in cities, as most older people in South Korea live in rural areas. Hypertension and diabetes are typical chronic diseases, among which hypertension, in particular, is the most significant risk factor for cardiocerebrovascular disease, requiring continuous management [6, 7]. The prevalence of hypertension among adults aged 30 years and above in South Korea was approximately 30% as of 2020, and there are disparities in the incidence rate of hypertension by age, income, and region of residence [8]. The age-standardized hypertension prevalence rate by region is 18.6% in Seoul, but Gangwon-do has the highest prevalence rate at 22.0%, which is a big difference. In addition, compared by area of residence, the number of age-standardized hospitalized hypertension patients per 1,000 population was 19.9 in Seoul. Jeollanam-do province had the highest number of age-standardized hospitalized hypertension patients and had 32.8, 50% more than Seoul (Supplementary Table S2).

In particular, some aspects of cardiocerebrovascular disease can cause emergencies that must be treated within the golden hour; hence, the local medical infrastructure plays an important role in this regard [9]. However, South Korea has an unbalanced medical infrastructure, resulting in a lack of response to medical demands within specific timelines. Looking at the average distance to the clinic by region, Seoul was 0.97 km, while Gangwon-do, which was the farthest, averaged 11.05 km, a huge difference (Supplementary Table S1). Such medical infrastructure is creating regional health disparities. However, hypertension, which is the most common risk factor for cardiocerebrovascular disease, is a typical ambulatory care sensitivity condition (ACSC), and the occurrence and exacerbation of complications can be prevented if it is managed continuously before it worsens [10]. The prognosis of the disease may vary depending on the consistency and adequacy of care provided at primary healthcare facilities in the community. Therefore, there is a need to develop a system that can effectively manage hypertension in rural areas.

The World Health Organization (WHO) places great importance on the management of chronic diseases that are closely related to human aging. Therefore, the WHO has indicated the need to maintain continuity of care (COC) in primary healthcare facilities to effectively manage chronic diseases [11]. The COC is one of the critical elements of primary care and represents a terminating and lasting relationship between healthcare providers and patients [12]. Previous studies have found that improving COC in local primary care settings to manage chronic diseases can effectively reduce avoidable hospitalizations and deaths [11, 13], there were also differences in COC depending on the regional scale [14]. Hence, COC is the most important factor in primary care, and improving this indicator will not only improve the health of the people but also help reduce regional health disparities. However, there is a paucity of research on the state of COC and regional health disparities. This study aims to explore regional health disparities in hypertension-related hospitalizations and confirm this difference according to the states of COC. Thus, we present data that has the potential to provide an effective basis for future policies aiming to resolve regional health disparities.

## Methods

### Data

Data were collected from the National Health Insurance Service National Sample Cohort (NHIS-NSC). South Korea introduced NHIS in 1977 to achieve universal medical coverage. The number of eligible people was gradually expanded, and in 1989 all citizens were covered [15]. NHIS covered 97% all citizens who reside in South Korea except medical aid beneficiaries, and healthcare beneficiaries for veterans [16]. The NHIS-NSC stores medical claims data of the entire Korean population. After stratifying the cohort into 1,476 strata by sex, age, type of insurance, and region, we randomly selected the target population and collected data equivalent to approximately 2% of the total population [17].

**FIGURE 1:**
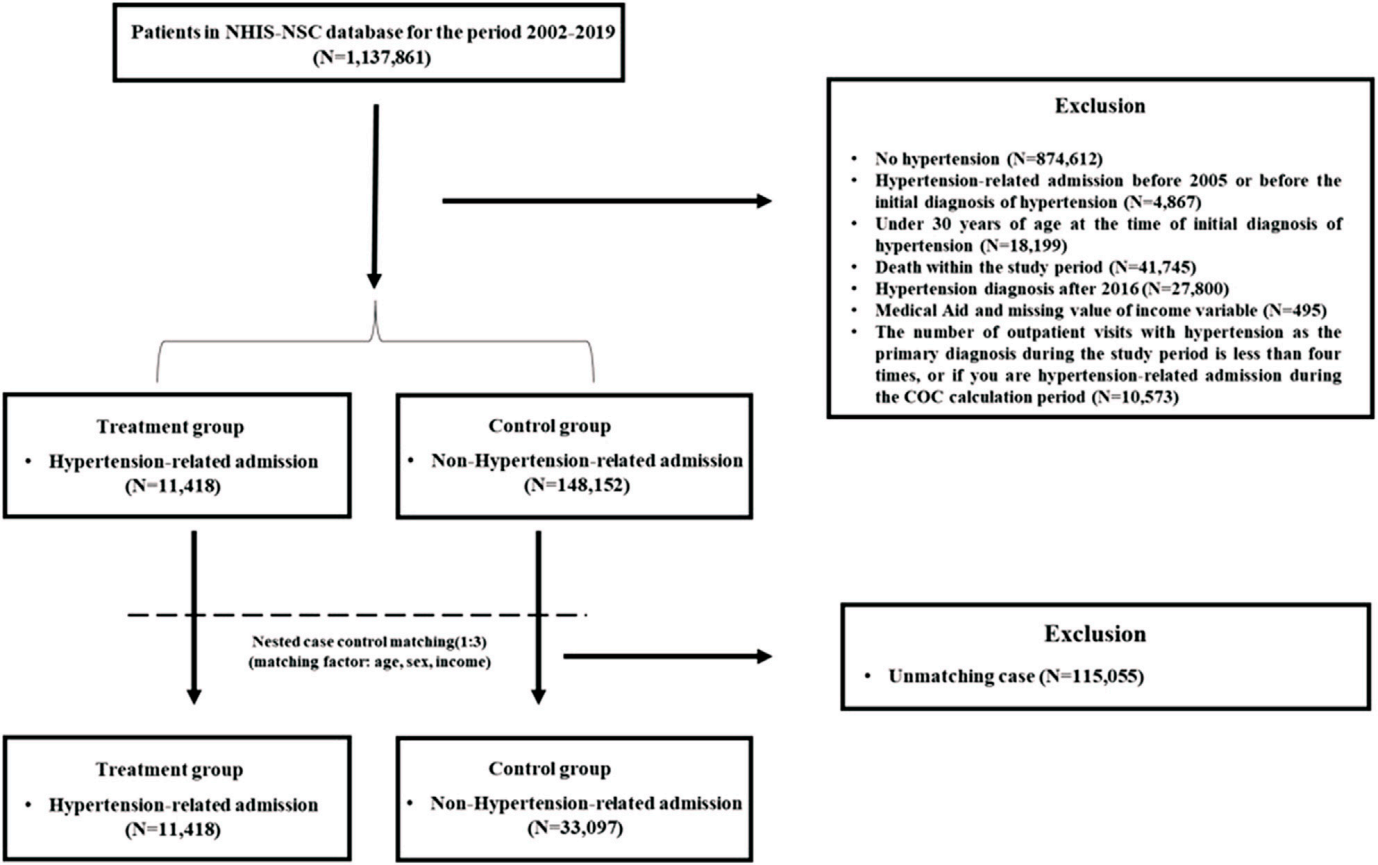
Study population selection process (South Korea, 2002–2019).

We collected data from 2002 to 2019. Of the 1,137,861 people, the study excluded those with no diagnosis of essential hypertension (I10). In addition, the patients who were admitted according to principal diagnosis for hypertension-related diseases (I10-I13, I15, I20-I25, I60-I69) according to the International Classification of Diseases (ICD-10) before 2005 or before the initial diagnosis of hypertension. Patients aged under 30 years, and those receiving medical aid were excluded from the study. In addition, we excluded hypertension patients who died to reduce possible competing risk from death and to reduce the bias of study results due to underlying health severity. The patients diagnosed with hypertension after 2016 were excluded from the analysis because the COC could not be observed for up to 3 years after 2016. In addition, the patients who visited the outpatient clinic fewer than four times during the analysis period and those who were admitted for hypertension-related diseases during the COC calculation period were excluded from the study. Thereafter, nested case-control (NCC) matching was performed with patients who had been admitted for hypertension-related disease as the treatment group and those who had never been admitted for hypertension-related disease as the control group. A total of 44,519 participants were included in the final analysis, excluding those who were dropped from the matching (Figure 1).

All statistical analyses were performed using the SAS statistical software (version 9.4; Cary, NC, United States). The need for ethical approval was waived by the Institutional Review Board of Yonsei University (1041849-202107-SB-107-01) because this study used only secondary data, and all personal information was anonymized and encrypted.

### Study Variables

#### Dependent Variable

Hypertension is a major risk factor for ischemic heart disease and cerebrovascular disease [18–20]. Therefore, our study defined the dependent variable “hypertension-related hospitalization” as hospitalization for hypertensive disease (I10-I13, I15), ischemic heart disease (I20-I25), or cerebrovascular disease (I60-I69). The principal diagnoses of hypertensive disease, ischemic heart disease, and cerebrovascular disease were confirmed according to the ICD-10 codes. At least one event of hypertension-related hospitalization from 2005 to 2019 was divided “Yes” or “No.”

#### Independent Variables

Based on the area of residence of study participants, the capital area (Seoul, Gionggi-do) and six metropolitan cities (Incheon-si, Daejeon-si, Gwangju-si, Daegu-si, Ulsan-si, Busan-si) were divided into urban. All other regions were divided into rural. South Korea is divided into -do, -si, -gun, and -gu according to the size of the administrative district. However, in the case of metropolitan cities, despite being in -si units, they allow for their own administrative district status given the city’s size and infrastructure. In South Korea, many infrastructures such as medical care, transportation, and facilities are concentrated in the capital area and the metropolitan city. In this study, we classified cities and rural areas in consideration of these administrative scales and infrastructures.

#### Control Variables

Control variables included the sociodemographic factors of age, sex, and income, as well as health-related factors of disability severity, CCI score (Charlson comorbidity index), and COC. Age was divided into units of 5 years from 30 to 64 years, with the addition of ≥65 years as a separate group, resulting in eight age groups. Income was divided according to quintiles: individual NHI premiums of 20% or less (quintile 1), 40% or less (quintile 2), 60% or less (quintile 3), 80% or less (quintile 4), 100% or less (quintile 5). Disabilities were categorized into grades 1–6, with grades 1–2 being severe, and grades 3–6 being mild disability [21]. The CCI was used as a representative index for adjusting for comorbidities. A weight of 0–6 was assigned to each disease according to Quan’s criteria, and the scores were divided into 0, 1, 2, 3, and above [22]. The COC is considered a reliable measure because it is used in the absence of routine visits by healthcare providers and is less sensitive to the number of visits by healthcare providers [23]. The COC indicator has the advantage of considering the number of healthcare providers and the number of visits together [12]. The COC has a value between 0 and 1, and the closer it is to 1, the better the COC [23]. Examples of COC scores by outpatient visits are present in Supplementary Figure S1. In this study, COC was calculated separately for 1, 2, and 3 years before the hypertension-related hospitalization. The COC was divided into the good COC group (COC = 1) and the poor COC group (COC < 1). The index was calculated using outpatient visits for essential hypertension (I10) using the following formula:

$$COC=\frac{\overset{M}{\underset{j=1}{\sum }}n_{j}^{2}-N}{N\left(N-1\right)}$$

where *N* is the total number of outpatient visits, *M* is the number of healthcare providers, and *n*_*j*_ is the number of visits to the *j*th healthcare provider [23].

### Statistical Analysis

The NCC study refers to a method in which an event occurring case is categorized into a treatment group, and participants with similar characteristics are extracted into control groups when the event occurs [24]. In this study, participants who experienced hypertension-related hospitalization were regarded as the treatment group, and a control group with similar characteristics was selected according to age, sex, and income, and 1:3 matching was performed.

Analysis was performed using the conditional logistic regression method, which is suitable for matched data [25]. Analysis was performed according to the COC observation period (1, 2, 3 years) with participants whose principal diagnoses were based on the ICD-10 codes for essential hypertension between 2002 and 2016. A stratified analysis of COC was performed to identify regional health disparities according to the states of COC. Using the COC criterion of 1, stratification analysis proceeded separately for the good COC group and the poor COC group. In addition, sensitivity analysis was performed by changing the COC criterion to 0.75.

## Results

The hypertension-related hospitalization fractions for the patients living in urban and rural areas were 24.1% and 28.4%, respectively. The fraction of hypertension-related hospitalizations was high in rural areas. The fraction of hypertension-related hospitalizations of patients with poor COC was 33.8%. Sex and income were used as matching variables, so there was no significant difference with regard to these variables. In the case of age, there was a difference in the hypertension-related hospitalizations fractions between the 30–34 and 35–39 groups. Furthermore, the more severe the disability and the higher the CCI score, the higher the hypertension-related hospitalizations fraction (Table 1).

**TABLE 1:** General characteristics (South Korea, 2002–2019).

Variable		Hypertension-related hospitalization			
		Yes (N = 11,418)	No (N = 33,097)	Total (N = 44,515)	*p*-value
		N (%)	N (%)	N (%)	
Region	Urban	6,950 (24.1)	21,846 (75.9)	28,796 (100.0)	<0.001
	Rural	4,468 (28.4)	11,251 (71.6)	15,719 (100.0)	
COC	Poor (<1)	3,507 (33.8)	6,856 (66.2)	10,363 (100.0)	<0.001
	Good (=1)	7,911 (23.2)	26,241 (76.8)	34,152 (100.0)	
Sex	Male	5,299 (26.0)	15,045 (74.0)	20,344 (100.0)	0.08
	Female	6,119 (25.3)	18,052 (74.7)	24,171 (100.0)	
Age	30 to 34	95 (42.0)	131 (58.0)	226 (100.0)	<0.001
	35 to 39	318 (30.4)	728 (69.6)	1,046 (100.0)	
	40 to 44	714 (26.7)	1,956 (73.3)	2,670 (100.0)	
	45 to 49	1,343 (25.5)	3,929 (74.5)	5,272 (100.0)	
	50 to 54	1,752 (25.0)	5,247 (75.0)	6,999 (100.0)	
	55 to 59	1,917 (25.3)	5,667 (74.7)	7,584 (100.0)	
	60 to 64	2,150 (25.0)	6,439 (75.0)	8,589 (100.0)	
	≥65	3,129 (25.8)	9,000 (74.2)	12,129 (100.0)	
Income	Quintile 1	2,240 (25.7)	6,487 (74.3)	8,727 (100.0)	0.249
	Quintile 2	1,589 (25.8)	4,572 (74.2)	6,161 (100.0)	
	Quintile 3	2,053 (25.3)	6,070 (74.7)	8,123 (100.0)	
	Quintile 4	2,471 (25.0)	7,394 (75.0)	9,865 (100.0)	
	Quintile 5	3,065 (26.3)	8,574 (73.7)	11,639 (100.0)	
Disability	Normal	10,646 (25.3)	31,352 (74.7)	41,998 (100.0)	<0.001
	Mild	492 (28.9)	1,210 (71.1)	1,702 (100.0)	
	Severe	280 (34.4)	535 (65.6)	815 (100.0)	
CCI score	0	8,936 (24.9)	26,913 (75.1)	35,849 (100.0)	<0.001
	1	1,551 (28.5)	3,888 (71.5)	5,439 (100.0)	
	2	759 (28.1)	1,943 (71.9)	2,702 (100.0)	
	≥3	172 (32.8)	353 (67.2)	525 (100.0)	

COC, continuity of care; CCI, charlson comorbidity index.

All study participants were divided into COC observation periods of 1, 2, and 3 years, and conditional logistic regression was performed (Table 2); rural areas were found to have higher ORs of hypertension-related hospitalization at 1.24 (95% CI, 1.20–1.31), 1.26 (95% CI, 1.20–1.32), and 1.25 (95% CI, 1.19–1.32), respectively, and poor COC was related to lower ORs than good COC for hypertension-related hospitalization of 0.59 (95% CI, 0.56–0.62), 0.67 (95% CI, 0.64–0.70), 0.23 (95% CI, 0.22–0.25), respectively.

**TABLE 2:** Results of conditional logistic regression (South Korea, 2002–2019).

Variable		Hypertension-related hospitalization											
		COC 1 year^a^				COC 2 years^a^				COC 3 years^a^			
		Crude		Adjusted		Crude		Adjusted		Crude		Adjusted	
		OR	95% CI	OR	95% CI	OR	95% CI	OR	95% CI	OR	95% CI	OR	95% CI
Region	Urban	1.00	—	1.00	—	1.00	—	1.00	—	1.00	—	1.00	—
	Rural	1.26	(1.20–1.31)	1.24	(1.19–1.30)	1.27	(1.21–1.33)	1.26	(1.20–1.32)	1.28	(1.21–1.34)	1.25	(1.19–1.32)
COC	Poor (<1)	1.00	—	1.00	—	1.00	—	1.00	—	1.00	—	1.00	—
	Good (=1)	0.59	(0.56–0.62)	0.59	(0.56–0.62)	0.67	(0.64–0.70)	0.67	(0.64–0.70)	0.23	(0.22–0.24)	0.23	(0.22–0.25)
Disability	Normal	1.00	—	1.00	—	1.00	—	1.00	—	1.00	—	1.00	—
	Mild	1.20	(1.08–1.34)	1.18	(1.06–1.32)	1.27	(1.13–1.43)	1.24	(1.10–1.39)	1.30	(1.15–1.48)	1.23	(1.08–1.40)
	Severe	1.54	(1.33–1.78)	1.50	(1.29–1.74)	1.70	(1.45–1.99)	1.65	(1.41–1.94)	1.65	(1.39–1.96)	1.63	(1.36–1.96)
CCI Score	0	1.00	—	1.00	—	1.00	—	1.00	—	1.00	—	1.00	—
	1	1.22	(1.14–1.30)	1.21	(1.13–1.29)	1.24	(1.16–1.32)	1.23	(1.15–1.32)	1.19	(1.11–1.28)	1.18	(1.09–1.28)
	2	1.17	(1.08–1.28)	1.16	(1.06–1.27)	1.16	(1.06–1.27)	1.14	(1.04–1.25)	1.14	(1.03–1.26)	1.09	(0.98–1.21)
	≥3	1.49	(1.24–1.79)	1.44	(1.19–1.73)	1.29	(1.06–1.57)	1.24	(1.02–1.51)	1.45	(1.17–1.80)	1.37	(1.08–1.73)

^a^ Adjusted for matching factors (age, sex, income), disability, and CCI score.

COC, continuity of care; OR, odds ratio; CI, confidence interval; CCI, charlson comorbidity index.

The results in Table 2 confirm that the odds of hypertension-related hospitalization differed according to the COC. Hence, a stratified analysis was performed by dividing the participants into good COC (COC = 1) and poor COC groups (COC < 1) (Table 3). In the good COC group, rural residence was associated with higher ORs of hypertension-related hospitalization at 1.19 (95% CI, 1.13–1.26), 1.15 (95% CI, 1.08–1.22), and 1.16 (95% CI, 1.08–1.25) than urban residence in the COC observation periods of 1, 2, and 3 years, respectively, and in the poor COC group, rural residence was associated with higher ORs at 1.48 (95% CI, 1.36–1.61), 1.44 (95% CI, 1.33–1.55), and 1.35 (95% CI, 1.26–1.45), respectively.

**TABLE 3:** Results of subgroup conditional logistic regression (South Korea, 2002–2019).

Scenario^a^ ^,^ ^b^		Hypertension-related hospitalization											
		COC 1 year				COC 2 years				COC 3 years			
		Crude		Adjusted		Crude		Adjusted		Crude		Adjusted	
		OR	95% CI	OR	95% CI	OR	95% CI	OR	95% CI	OR	95% CI	OR	95% CI
Total	Region												
	Urban	1.00		1.00		1.00		1.00		1.00		1.00	
	Rural	1.26	(1.20–1.31)	1.24	(1.19–1.30)	1.27	(1.21–1.33)	1.26	(1.20–1.32)	1.28	(1.21–1.34)	1.25	(1.19–1.32)
COC classification													
Good (=1)	Urban	1.00		1.00		1.00		1.00		1.00		1.00	
	Rural	1.19	(1.13–1.26)	1.19	(1.13–1.26)	1.15	(1.08–1.23)	1.15	(1.08–1.22)	1.17	(1.09–1.25)	1.16	(1.08–1.25)
Poor (<1)	Urban	1.00		1.00		1.00		1.00		1.00		1.00	
	Rural	1.49	(1.37–1.62)	1.48	(1.36–1.61)	1.45	(1.34–1.56)	1.44	(1.33–1.55)	1.36	(1.27–1.46)	1.35	(1.26–1.45)
^c^COC classification (by 0.75)													
Good (≥0.75)	Urban	1.00		1.00		1.00		1.00		1.00		1.00	
	Rural	1.23	(1.17–1.30)	1.23	(1.17–1.29)	1.18	(1.12–1.25)	1.18	(1.11–1.24)	1.22	(1.15–1.30)	1.22	(1.15–1.29)
Poor (<0.75)	Urban	1.00		1.00		1.00		1.00		1.00		1.00	
	Rural	1.55	(1.40–1.72)	1.54	(1.39–1.70)	1.57	(1.40–1.76)	1.56	(1.39–1.75)	1.41	(1.27–1.57)	1.41	(1.27–1.56)

a Adjusted for matching factors (age, sex, income), disability, and CCI score.

^b^ All scenarios are the result of comparing metropolitan (reference) and rural areas.

^c^ Sensitivity analysis.

COC, continuity of care; OR, odds ratio; CI, confidence interval.

Sensitivity analysis was performed by changing the COC criterion to 0.75 (Table 3). In the good COC group (COC ≥ 0.75), rural residence was associated with higher ORs of hypertension-related hospitalization at 1.23 (95% CI, 1.17–1.29), 1.18 (95% CI, 1.11–1.24), and 1.22 (95% CI, 1.15–1.29) than urban residence in the COC observation periods of 1, 2, and 3 years, respectively, and in the poor COC group (COC < 0.75), rural residence was associated with higher ORs at 1.54 (95% CI, 1.39–1.70), 1.56 (95% CI, 1.39–1.75), and 1.41 (95% CI, 1.27–1.56), respectively, than urban residence. The results of the analysis confirmed that the odds of hypertension-related hospitalization were higher among participants living in rural areas than among those living in urban areas. In addition, there was a difference in the odds of hypertension-related hospitalization between urban and rural areas due to COC.

## Discussion

### Principal Results

We investigated the factors related to the occurrence of hypertension-related hospitalizations of hypertensive patients, focusing on regional health disparities, using nationally representative medical service claims data. In addition, we confirmed the health disparity in regions according to the maintenance of the continuity of outpatient medical care. Our analysis using NCC matching and conditional logistic regression revealed that the odds of hypertension-related hospitalization were significantly higher in rural areas than in metropolitan areas. This trend was consistent, regardless of the state or calculation period of COC.

### Interpretation

Hypertension is one of the most common chronic diseases, and hypertension-related hospitalization can be prevented. Avoidable or preventable hospitalization refers to hospitalization that occurs because adequate primary care is not provided [26], and it has been used as an indicator to identify health inequality in several studies [27–29]. Although hypertension is a primary underlying cardiovascular disease, aggravation of the disease can be prevented through ongoing management [10].

When people achieve a certain level of health through the national medical system and policies, the government’s next task is to solve health inequalities. For example, a statistically significant relationship between racial and ethnic characteristics and preventable hospitalization has been observed in the United States [30]. One of the most prominent issues in South Korea is health inequality between urban and rural regions [31]. According to a study by the Korea Centers for Disease Control and Prevention (KCDC), the regional disparity in the treatment rates of hypertension and diabetes patients has been increasing over the past 3 years [19–21] [32]. There was a statistically significant relationship between residential area and the occurrence of hypertension-related hospitalization, even when other conditions were sufficiently controlled. This result is similar to that of other studies [33, 34] that compared preventable hospitalization in rural and urban areas. The disparity may be the result of physical access to medical facilities, socioeconomic development of a given region, and differences in demographic structure [35, 36].

In addition, this study confirms the results of many previous studies [37–40], wherein a high COC for chronic diseases was strongly associated with a decrease in avoidable hospitalization. Those with a COC value of 1 had lower odds of hypertension-related hospitalization than those with a COC value <1. This trend was maximized when the COC calculation period was 3 years. The occurrence of avoidable hospitalization entails social and economic losses. In a study conducted in Portugal, the average estimated cost per avoidable hospitalization was €2,515 [41].

The Korean government implemented the primary healthcare chronic disease management pilot project in January 2019 after implementing the community-based hypertension and diabetes registry program, a chronic disease management program at the clinical level, a community-based primary care project, and a pilot project for reimbursing chronic disease care [42]. The primary healthcare chronic disease management pilot project aimed to ensure continuous management of patients with high blood pressure and diabetes who visited neighborhood clinics and is in the process of converting to a main project. Several studies have shown that primary care-centered chronic disease management is cost-effective and improves COC and medication adherence [43–46]. To improve COC, it is important to introduce policies at the national level and make steady efforts to manage health at the individual level.

On the other hand, subgroup analysis revealed the possibility of a high COC reducing the health disparity between the regions. We found that the difference in interregional hypertension-related hospitalization was significant even in the group with a high COC. However, the OR of the residential area variable tended to be lower in the group with COC values closer to 1. This trend implies that a high COC may reduce regional disparities. These results are meaningful because they can be used as evidence for asserting the importance of improving the COC.

### Limitations

This study had several limitations. First, the matching rate may have been low because of the misclassification of codes in the claim data. However, since we confirmed the hospitalization for hypertension and cardiovascular disease, and as cardiovascular disease trends are being monitored by the National Health Insurance (NHI) and Health Insurance Review & Assessment Service (HIRA), we believe that the code mismatch rate in our study is low [47]. Second, individual health behaviors and regional medical resources could not be considered as variables due to data limitations. Some studies have reported differences in health behaviors between regions, such as drinking, smoking, and regular walking [48, 49], and differences in medical resource allocation that can eventually lead to health disparities [50, 51]. These findings suggest the need for detailed research in the future. Third, through the results of this study, it was confirmed that regional health disparities occur due to differences in the quality of primary care, but other causes could not be determined. Regional health disparities are affected by underlying factors such as differences in healthcare infrastructure, aging populations, and the environment. Therefore, future studies are needed to confirm the impact in addition to primary care. Fourth, hospitalization for other diseases in hypertensive patients may affect hypertension-related hospitalizations, but this study did not consider these effects. However, we tried to control for this effect by adjusting for the health-related variable (disability, and CCI).

### Conclusion

The odds of hypertension-related hospitalizations were significantly higher in rural residents than in urban residents, regardless of the state of COC and observation period. These results can be viewed as evidence of regional health disparities. Although it was confirmed that regional health disparities were somewhat resolved through the enhancement of COC, regional health disparities still existed due to differences in the quality of primary care. Therefore, to reduce regional health disparities, both the promotion of COC and the improvement of the quality of primary care must be achieved.
