# Supplementary material for: Regional Health Disparities in Hypertension-Related Hospitalization of Hypertensive Patients: A Nationwide Population-Based Nested Case-Control Study
Source: Int J Public Health. 2023 Jan 24;68:1605495. doi: 10.3389/ijph.2023.1605495 (PMC9902356; doi:10.3389/ijph.2023.1605495)
Supplement: Supplementary file 1 [file DataSheet1.docx]

Supplementary Table 1. Number of healthcare facilities and access to healthcare facilities by region (South Korea, 2002-2019)

| Region | | ^a^Population | ^a^Elderly rate  (≥65) | Clinic | | Hospital | | | General Hospital | | |  |
| --- | --- | --- | --- | --- | --- | --- | --- | --- | --- | --- | --- | --- |
|  |  |  |  | ^b^Number  of clinic | ^c^Accessibility (km) | | ^b^Number  of hospital | ^c^Accessibility (km) | | ^b^Number of  general hospital | ^c^Accessibility (km) | |
| Capital area | Seoul | 9,436,836 | 17.5 | 9,189 | 0.97 | | 222 | 1.97 | | 56 | 2.85 | |
|  | Gyeonggi-do | 13,583,238 | 14.6 | 7,487 | 4.71 | | 277 | 9.30 | | 67 | 16.54 | |
| Metropolitan city | Busan-si | 3,320,276 | 21.4 | 2,468 | 2.80 | | 136 | 4.38 | | 28 | 6.80 | |
|  | Daegue-si | 2,365,619 | 18.3 | 1,884 | 3.79 | | 88 | 5.71 | | 18 | 9.70 | |
|  | Incheon-si | 2,964,820 | 15.6 | 1,633 | 5.90 | | 56 | 8.72 | | 20 | 11.55 | |
|  | Gwangju-si | 1,432,049 | 15.5 | 970 | 2.69 | | 84 | 4.86 | | 23 | 6.04 | |
|  | Daejeon-si | 1,446,749 | 16.0 | 1,112 | 3.25 | | 46 | 5.43 | | 10 | 6.76 | |
|  | Ulsan-si | 1,111,371 | 14.7 | 618 | 4.81 | | 33 | 5.94 | | 9 | 9.29 | |
| Rural | Sejong-si | 382,770 | 10.4 | 198 | 5.12 | | 1 | 13.08 | | 2 | 11.09 | |
|  | Gangwon-do | 1,536,863 | 22.7 | 782 | 11.05 | | 37 | 22.73 | | 15 | 30.47 | |
|  | Chungcheongbuk-do | 1,595,284 | 19.8 | 912 | 7.81 | | 37 | 14.56 | | 13 | 22.75 | |
|  | Chungcheongnam-do | 2,122,357 | 20.5 | 1,094 | 6.42 | | 46 | 15.76 | | 13 | 18.61 | |
|  | Jeollabuk-do | 1,770,839 | 23.1 | 1,179 | 6.64 | | 64 | 13.60 | | 13 | 21.84 | |
|  | Jeollanam-do | 1,818,484 | 25.1 | 962 | 6.11 | | 74 | 14.00 | | 25 | 21.89 | |
|  | Gyeongsangbuk-do | 2,602,052 | 23.7 | 1,279 | 9.12 | | 55 | 15.83 | | 20 | 26.85 | |
|  | Gyeongsangnam-do | 3,282,849 | 19.4 | 1,682 | 7.16 | | 134 | 12.54 | | 26 | 31.54 | |
|  | Jeju-island | 678,373 | 17.0 | 463 | 4.35 | | 7 | 20.98 | | 6 | 22.29 | |

^a^Statistics Korea. Elderly population rate. (2022) Available from: https://kosis.kr/statHtml/statHtml.do?orgId=101&tblId=DT_1YL20631&conn_path=I2 (Accessed Dec 23, 2022)

^b^Statistics Korea. Current status of healthcare facilities by region 2021. (2021) Available from: https://kosis.kr/statHtml/statHtml.do?orgId=350&tblId=TX_35003_A002&vw_cd=MT_ZTITLE&list_id=&scrId=&seqNo=&lang_mode=ko&obj_var_id=&itm_id=&conn_path=E1&docId=00650&markType=S&itmNm=%EC%A0%84%EA%B5%AD (Accessed Dec 23, 2022)

^c^Ministry of Land, Infrastructure and Transport. Land monitoring report 2020. (2021) Available from: https://policy.nl.go.kr/search/searchDetail.do?rec_key=SH2_PLC20210269681 (Accessed Dec 23, 2022)

| Region | | ^a^ Age-standardized hypertension prevalence | ^b^ Age-standardized hypertension  hospitalization  (per 1,000) |
| --- | --- | --- | --- |
|  |  |  |  |
| Capital area | Seoul | 18.6 | 19.9 |
|  | Gyeonggi-do | 20.1 | 21.8 |
| Metropolitan city | Busan-si | 17.9 | 30.4 |
|  | Daegue-si | 18.1 | 23.5 |
|  | Incheon-si | 22.3 | 24.8 |
|  | Gwangju-si | 17.1 | 29.7 |
|  | Daejeon-si | 19.5 | 26.4 |
|  | Ulsan-si | 18.6 | 29.4 |
| Rural | Sejong-si | 20.5 | 24.0 |
|  | Gangwon-do | 22.0 | 25.5 |
|  | Chungcheongbuk-do | 20.5 | 25.0 |
|  | Chungcheongnam-do | 21.5 | 25.4 |
|  | Jeollabuk-do | 19.9 | 29.6 |
|  | Jeollanam-do | 18.2 | 32.8 |
|  | Gyeongsangbuk-do | 19.1 | 25.7 |
|  | Gyeongsangnam-do | 18.7 | 30.1 |
|  | Jeju-island | 18.6 | 18.7 |

Supplementary Table 2. Hypertension prevalence and hospitalization by region (South Korea, 2002-2019)

^a^Statistics Korea. Hypertension Prevalence by region (over 30 age). (2021). Available from: https://kosis.kr/statHtml/statHtml.do?orgId=177&tblId=DT_HYPER_DOCTOR&vw_cd=MT_ZTITLE&list_id=F_001_002_001&seqNo=&lang_mode=ko&language=kor&obj_var_id=&itm_id=&conn_path=MT_ZTITLE (Accessed Jan 2, 2023)

^b^Korea National Medical Center. Statistics of public healthcare in 2019. (2020). Available from: https://www.nmc.or.kr/nmc/bbs/B0000058/view.do?nttId=586&menuNo=200326&pageIndex=1 (Accessed Dec 23, 2022)


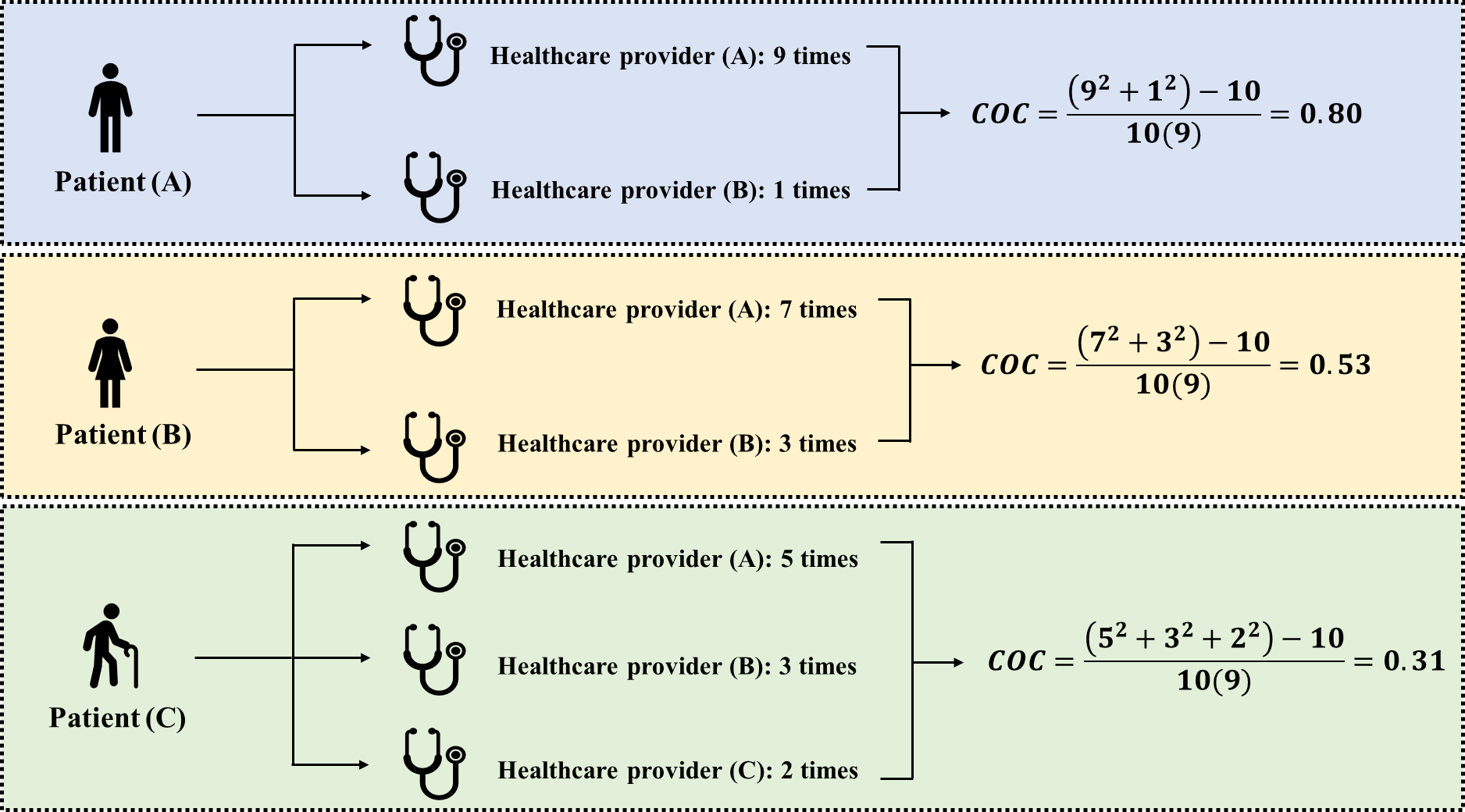


Supplementary Figure 1. Example of continuity of care scores by outpatient visits (South Korea, 2002-2019)
